# Serious games and blended learning; effects on performance and motivation in medical education

**DOI:** 10.1007/s40037-016-0320-2

**Published:** 2016-12-14

**Authors:** Mary Dankbaar

**Affiliations:** Institute of Medical Education Research Rotterdam (iMERR) and the Department of Education, Erasmus University Medical Center, Rotterdam, The Netherlands

**Keywords:** Serious games, Blended learning, Performance, Motivation, Cognitive skills

## Abstract

**Introduction:**

More efficient, flexible training models are needed in medical education. Information technology offers the tools to design and develop effective and more efficient training. The aims of this thesis were: 1) Compare the effectiveness of blended versus classroom training for the acquisition of knowledge; 2) Investigate the effectiveness and critical design features of serious games for performance improvement and motivation.

**Methods:**

Five empirical studies were conducted to answer the research questions and a descriptive study on an evaluation framework to assess serious games was performed.

**Results:**

The results of the research studies indicated that: 1) For knowledge acquisition, blended learning is equally effective and attractive for learners as classroom learning; 2) A serious game with realistic, interactive cases improved complex cognitive skills for residents, with limited self-study time. Although the same game was motivating for inexperienced medical students and stimulated them to study longer, it did not improve their cognitive skills, compared with what they learned from an instructional e‑module. This indicates an ‘expertise reversal effect’, where a rich learning environment is effective for experts, but may be contra-productive for novices (interaction of prior knowledge and complexity of format).

**Discussion:**

A blended design is equally effective and attractive as classroom training. Blended learning facilitates adaptation to the learners’ knowledge level, flexibility in time and scalability of learning. Games may support skills learning, provided task complexity matches the learner’s competency level. More design-based research is needed on the effects of task complexity and other design features on performance improvement, for both novices and experts.

## Introduction

In medical education, with its exponential growth in knowledge and increasing demands on the competencies required by doctors, there is a need for more efficient training models [1]. Blended and online learning are powerful learning concepts, which can be used in different formats. Blended learning may combine the advantages of online and classroom learning. Its use is increasing quickly and a precipitous growth is predicted [2]. The first aim of this thesis was to compare the effectiveness of a blended training design with classroom training for the acquisition of knowledge. One of the promising new online formats to train complex skills in a motivating way is provided by serious games. Little is yet known about the effectiveness and optimal design of serious games in health care training [3–6]. The second aim of this thesis was to investigate the effectiveness and critical design features of serious games for performance improvement and motivation. This thesis consists of five research studies and an evaluation framework for serious games [7].

## Methods

Study 1: In order to compare the effectiveness of a blended training design with classroom training, we performed a retrospective study and compared the effectiveness of a postgraduate course for nurses in a conventional classroom-based design (11-day course) with a blended course design, consisting of two-thirds classroom training (7 days) and one-third online self-study. Results on the knowledge tests and evaluation questionnaire were compared for both groups and costs of both types of postgraduate training for participating organizations were calculated.

Study 2: Before investigating the effectiveness of a serious game in emergency care (abcde*SIM*), we performed a validation study on commonly used formats, both nationally and internationally, in the assessment of emergency care skills for residents. A psychometric analysis of a checklist, a competency scale and a global performance scale were conducted. The validity and inter-rater reliability of the assessment instrument in a certified training for residents were evaluated, using video-taped assessments.

Study 3: We investigated whether residents, who used a serious game (abcde*SIM) *as an additional preparation for classroom training, showed better cognitive skills before training than residents who only used a course manual as a preparation. In a quasi-experimental design, with residents preparing for a rotation in the emergency department, one group received a course manual as preparation for face-to-face training and another group additionally received access to the serious game as a preparation for the same training. Emergency care skills were assessed in a scenario assessment with simulation patient, using the validated assessment instruments resulting from study 2.

Study 4: As the previous study was inconclusive on the design choices that were responsible for the performance improvement, in this study we focused on the question of what the effects are of adding high-fidelity cases (a simulation game) versus low fidelity (text-based) cases to an instructional e‑module on the cognitive skills and motivation of fourth-year medical students. We set up a three-group randomized design: a control group working on an e‑module; a cases group, combining the e‑module with low-fidelity text-based patient cases; and a game group, combining the e‑module with a high-fidelity simulation game, with the same cases. Cognitive load and motivation were evaluated after the intervention. After a 2-week study period, assessors (who were blinded for the condition) rated students’ cognitive emergency care skills in two mannequin-based scenarios, using the assessment instruments resulting from study 2.

Study 5: The fifth study focused on the question of whether undergraduate medical students develop better patient safety knowledge and awareness and are more motivated after using a serious game (Air Medic Sky-1), typically highly interactive, or after using an e‑module, typically less interactive, on the same patient safety topics. Fourth-year medical students were randomly assigned to either a serious game, containing video lectures and patient missions, or an e‑module, containing text-based lectures on the same topics. A third group acted as a historical control group without extra instruction. Students were tested on knowledge, self-efficacy and motivation. During their clinical rotation they reported perceived stress and patient-safety awareness on a weekly basis.

## Results

1. This study indicates that for knowledge acquisition, a blended course design is equally effective and attractive as a classroom-based course design. In postgraduate training, a significant reduction in training costs can be achieved with blended learning, without compromising performance [8].
2. For the assessment of complex cognitive emergency care skills, checklists have poor validity and reliability compared with more global competency scales or rating scales [9].
3. A serious game on emergency care with realistic, interactive cases improved complex cognitive skills for residents, despite limited self-study time [10].
4. Although the same game was motivating for inexperienced medical students and stimulated them to study longer, it did not improve their cognitive skills, compared with what they learned from an instructional e‑module [11].
5. Our study on a game in patient safety indicates that video-lectures (in a game) and text-based lectures (in an e‑module) are equally effective in developing knowledge on patient safety. Although serious games with interactive patient cases are strongly engaging for students and stimulate them to study longer, they do not necessarily result in better performance in patient safety issues [12].

## Discussion

Our results that blended learning is as effective as classroom-based learning are consistent with systematic reviews in the general domain [13]. Blended learning enables adaptation to the learner’s knowledge level, flexibility in time and scalability of learning. An important consideration in a blended design is securing online preparation by students. Our combined results on a serious game in emergency care, where the game improved residents’ cognitive skills, but did not improve inexperienced students’ skills, indicate an ‘expertise reversal effect’ [14]: a rich learning environment, such as a highly interactive game, may benefit experts, but is counter-productive for novice learners (interaction between prior knowledge and complexity of format). Although serious games with realistic tasks often enhance motivation, they are as such not sufficient for improving performance. Games may support skills learning, provided task complexity matches the learner’s competency level. Design-based research should shed more light on how game design may enhance skills learning. Another interesting field for further study is the question which game features enhance motivation? Features that are mentioned in the literature are: control and interaction, a story-line and challenges [15].

## Advice

One piece of advice for PhD students on how to successfully complete a PhD project:

Choose a subject and research focus which fits your passion and genuine interest. You will find PhD research is fun to do, at times challenging, but a rewarding learning experience.
